# Treating p53 Mutant Aggregation-Associated Cancer

**DOI:** 10.3390/cancers10060154

**Published:** 2018-05-23

**Authors:** Mathumai Kanapathipillai

**Affiliations:** Department of Mechanical Engineering, University of Michigan-Dearborn, Dearborn, MI 48128, USA; mathumai@umich.edu

**Keywords:** p53 mutant, aggregation, drugs, cancer, inhibition

## Abstract

p53 is a tumor suppressor protein. Under stressful conditions, p53 tightly regulates cell growth by promoting apoptosis and DNA repair. When p53 becomes mutated, it loses its function, resulting in abnormal cell proliferation and tumor progression. Depending on the p53 mutation, it has been shown to form aggregates leading to negative gain of function of the protein. p53 mutant associated aggregation has been observed in several cancer tissues and has been shown to promote tumor growth. Recent studies show correlation between p53 mutant aggregation, functional loss, and tumor growth. Moreover, p53 aggregation has been observed in biopsies, patient tissues, and in vivo studies. Given the fact that over fifty percent of cancers have p53 mutation and several of them are prone to aggregation, therapeutic strategies are needed for treating p53 mutant aggregation associated cancers. Recent studies using polyarginine analogues and designer peptides for inhibiting p53 aggregation and tumor growth gives further encouragement in treating cancer as a protein aggregation disease. In this review, we highlight the recent efforts in targeting p53 aggregation in cancer and propose the use of small stress molecules as potential p53-antiaggregation drugs.

## 1. Introduction

Many protein aggregation diseases are the result of protein misfolding/aggregation due to genetic mutations or environmental stress conditions [1]. Protein aggregation, instability, misfolding, or defective transport leads to disruption in cellular process and function [1,2]. Recent studies indicate that similar to neurodegenerative diseases, protein misfolding/aggregation is thought to be involved in some cancers [3,4]. In cancer, protein misfolding/aggregation affects mainly the p53 protein [3,5,6,7]. p53 is called a tumor suppressor protein as it controls the cell cycle, DNA replication, and uncontrolled cell division during tumor growth [8]. When the p53 protein is mutated or aggregated, it loses its function, resulting in tumor progression and growth.

**Figure 1:**
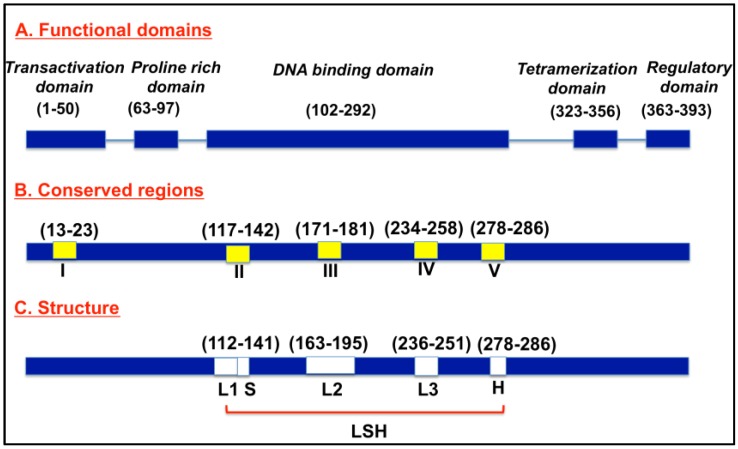
Schematic of p53 protein structure.

Normally, p53 proteins are switched off and are activated when cells experience stress and undergo uncontrolled division and proliferation [9,10]. When uncontrolled cell growth results, p53 induces p21 expression, leading to cell cycle arrest [11,12]. When damage is beyond repair, p53 triggers programmed cell death by triggering apoptosis-involved genes, including BAX, a proapoptotic member of the Bcl-2 family [13]. Figure 1 depicts a schematic representation of p53. The p53 protein consists of 393 amino acids, with four major functional domains: transcriptional, DNA binding, tetramerization, and regulatory domains [14]. The protein consists of five conserved regions (I, II, III, IV, V), and loop-helix structure (L, S, H) forming regions. The loop domains overlap with the highly conserved domains and are part of the three-dimensional structure of the protein. In addition, there is a strong correlation between the mutations and p53’s three-dimensional structural domains [14].

When p53 is mutated, it loses its function and becomes inactivated [15,16,17]. More often, p53 loses its function by a single point mutation, which leads to alterations in DNA binding capacity (contact mutant), or structural alterations of the core DNA binding domain of the protein (conformational mutants). Recent findings show that mutant p53 and its fragments can form protein aggregates in vitro and in vivo [18,19]. Both contact and conformational mutant p53 aggregation is observed in tumor tissue samples from patients, biopsies, and in several cancer cell lines, indicating the correlation between p53 mutant aggregation and tumor growth [15,18]. p53 hot spot mutants (both contact and conformational) at amino acid locations 175, 245, 248, 249, 273, and 282 are frequently observed in most cancers [20]. Among the hot spot mutants, R248Q, R248W, and R175H mutations have shown aggregation in various tumor samples, while R273H and R249S hot spot mutant-bearing tumor samples did not have aggregation [21,22]. Studies have further shown loss of p53 functionality with p53 aggregation. Moreover, studies show gain of function of p53 mutants by co-aggregation with p53 family proteins, resulting in dominant negative oncogenic activity of p53 [18], indicating the importance of treating p53 mutant associated aggregation. In addition, prion-like aggregation properties have been observed in mutant p53-bearing cancer cells, suggesting negative dominant effects of p53 mutant aggregates [16,23]. The above findings suggest stronger correlation between p53 mutant aggregation and tumor growth. Hence, there is a very clear need for finding therapeutics to target p53 mutants that result in aggregation, which subsequently leads to cancer progression. 

**Figure 2:**
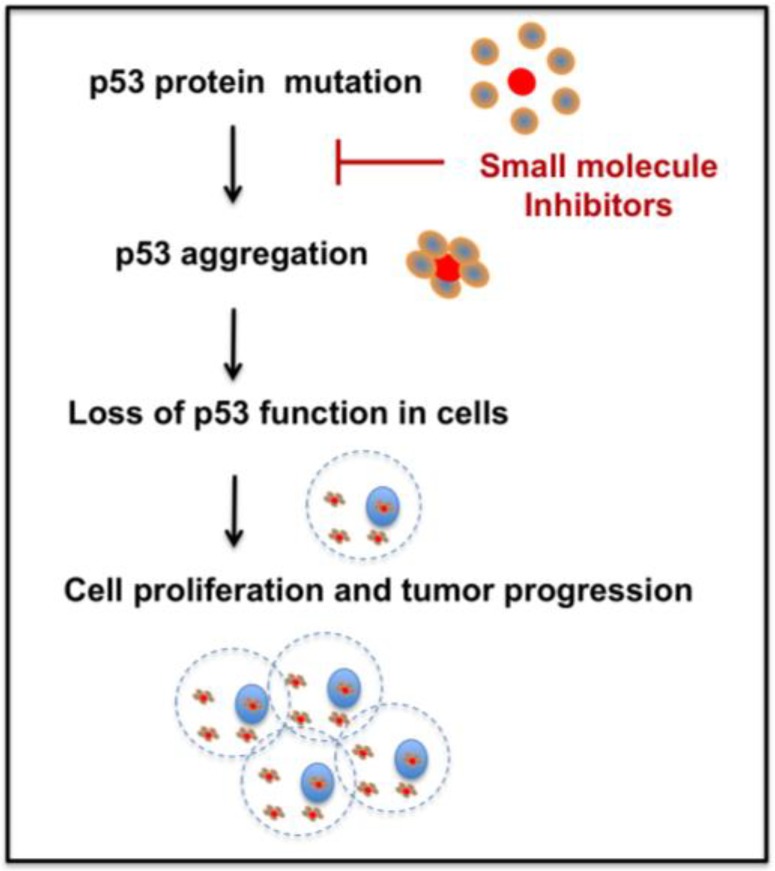
Schematic of the p53 mutant aggregation inhibition approach for treating cancer.

Several methods have emerged to rescue p53 mutant function [24,25]. Therapies to reactivate p53, restoring p53 downstream functions, and promoting mutant p53 degradation are gaining interest [24,26]. But, therapeutic strategies for p53 aggregation inhibition are relatively new and need further research. As recent evidence shows, mutant p53 aggregation plays a critical role in cancer and studying the aggregation tendency and the development of successful new drugs is important for cancer therapy. Hence, development of novel p53 mutant anti-aggregation drugs could open new directions in cancer therapy. Figure 2 depicts one potential approach of targeting p53 mutant aggregation and subsequent tumor growth utilizing small molecules. The following sections of the manuscript briefly discuss the recent therapeutic approaches to treat p53 mutant aggregation, and challenges for the future.

## 2. Therapeutic Approaches 

Treating cancer as a p53 aggregation disease is still in its early stages. Only a few studies have reported targeting cancer as a p53 mutant aggregation disease. We have shown that small stress molecules, such as polyarginine and its analogues, exhibit p53 aggregation inhibition both in vitro and in p53 mutant cancer cells [27,28]. Another recent study showed the potential of a designer peptide to inhibit p53 mutant aggregation and tumor growth in vivo [22]. Table 1 summarizes the therapeutic approaches. A brief summary of the studies is presented below. 

### 2.1. Small Stress Molecules 

Several small stress molecules have been shown to stabilize proteins under harsh conditions. In particular, compatible solutes have been shown to stabilize protein aggregation. The molecules have shown inhibitory potential in neurodegeneration caused by peptide aggregation [29,30,31,32]. In our previous studies, we have found that small stress molecules such as ectoine, hydroxyectoine, and mannosylglycerate, which are known to stabilize proteins under high-stress conditions, have proven to be effective in inhibiting neurodegenerative disease-causing peptides [32,33]. Interestingly, p53 mutant cancers may share a common aggregation mechanism with neurodegenerative diseases. Moreover, recent observations of prion aggregate-like behavior of p53 mutant aggregates [15] indicate the possibilities for similarities between the protein aggregation in neurodegenerative disease and cancer. Hence, small stress molecules could have the potential of modulating p53 mutant aggregation. Studies have shown the potential of arginine in protein disaggregation and stabilization [34,35,36,37]. Similar to arginine, its analogues ornithine, canavanine, and citrulline also could have the ability to stabilize proteins via a similar mechanism. 

Inspired by the above, we recently studied the ability of arginine molecules to modulate p53 aggregation and cancer cell proliferation, to identify therapeutic candidates for treating cancer due to p53 aggregation. We tested the inhibitory effects of polyarginine and its analogues on the R248Q p53 mutant mimetic peptide QRPILTIITL aggregation, and p53 mutant-bearing cancer cell growth in vitro. The study revealed that polyarginine and polyornithine significantly inhibit p53 mutant peptide aggregation in vitro and inhibit the growth of p53 mutant (R248Q) lung cancer cells H719 and p53 mutant (R175H) breast cancer cells SK-BR-3. The molecules showed no effect on p53 wildtype and p53 null cancer cell growth. In addition, the molecules enhanced p21 expression, one of the p53 target genes responsible for cell cycle arrest [12], indicating a potential role in functional restoration. Further, the molecules did not exhibit significant toxicity to normal cells at the relevant tested concentrations [28]. The studies need to be expanded to understand the modulation mechanism of arginine and its analogues on p53 aggregation. Further, the molecules could be delivered via nanodepots or a suitable drug carrier to increase its half-life, bioavailability, and targeting specificity, thus increasing the therapeutic efficacy. 

In addition to arginine and analogues, in a separate study, we have also shown that the cationic small stress molecule acetylcholine chloride had the ability to inhibit aggregation of p53 mutant peptide WRPILTIITL (mimicking the p53 hot spot mutant R248W) in vitro [27]. Acetylcholine has been shown to prevent osmotic stress in *Lactobacillus plantarum* [38], and is also reported to exhibit anticancer properties [39]. The above reported findings on the p53 mutant anti-aggregation properties of arginine, its analogues, and acetylcholine chloride indicate that small molecule protein stabilizers could have selective inhibitory effects towards aggregation associated with p53 mutants.

### 2.2. Designer Peptides

Recently, Soragni et al. [22] developed a peptide-based approach to inhibit p53 mutant aggregation and tumor growth. The designer peptide, coined as ReACp53, exhibited p53 mutant aggregation inhibition and tumor suppression in vitro and in vivo. The peptides were designed to inhibit the p53 aggregation-prone region 252–258. The designed peptide LTRITLE was then fused with arginine residues to facilitate cell penetration, and a 249–251 p53 residue (RPI) to yield the designer peptide ReACp53. The potential of the peptide in p53 mutant aggregation was tested in various ovarian cancer models in vitro and in vivo. The peptides were able to inhibit p53 aggregation, rescue p53 function, and inhibit tumor growth in vivo [22]. 

However, the method has its limitations as well. The authors noted that if the wildtype p53 is partially unfolded and aggregated, the designer peptide would probably target the wildtype p53 structures as well. Hence, there can be systemic toxicity effects if this happens in normal cells. In addition, the inhibitory ability is limited to certain p53 mutant aggregations and may not be applicable to all p53 aggregation-associated cancer. Further, like all small molecule-based therapies, the peptides will have short half-life in vivo and will be cleared from circulation rapidly, and to realize the full potential, further modifications and optimization may be needed. 

## 3. Challenges for the Future

The studies discussed in this review show that small molecules are capable of modulating p53 mutant aggregation and subsequent tumor progression. However due to their small molecular weight and size, they have a higher probability of getting rapidly cleared by the body, minimizing their potential as effective therapies. Hence, it would be ideal if the small stress molecules could be formulated in a stealth drug carrier, and to have target specificity to the tumor microenvironment to realize its full translational potential. Compared to conventional therapies which suffer from poor circulation, bioavailability, and efficacy, nanotechnology approaches can remedy some of the problems due to their tunable design [40]. Nanoparticles have the potential to deliver drug efficiently to the diseased site while sparing normal tissues. Further, nanoformulations could be tuned to have better bioavailability and plasma solubility, thereby increasing the therapeutic efficacy and half-life of drug molecules. Nanoformulations have been used as a major delivery system in cancer drug delivery [40,41]. Formulation strategies that are used in cancer drug delivery, including polymeric nanoparticles and liposomes, are advantageous due to their ability to incorporate the drugs in the core or on the surface/bilayer of the particles, depending on the drug properties. Hence a nanocarrier-based delivery approach of p53 anti-aggregates to specifically target p53 mutant cancer cells would minimize systemic side effects and could yield better therapeutic outcomes.

Finally, to further enhance the therapeutic potential, personalized medicine approaches could be utilized. To precisely target specific p53 mutant aggregation-associated cancer, mutant specific anti-aggregation targets could be developed. A similar concept to recent studies reported on p53 mutant antibodies to inhibit p53 mutants in cancer [42] could be applied for patient-specific p53 mutant aggregation targeting. To summarize, developing a small molecule formulation that could have the above-mentioned properties could be a great therapeutic approach to treat p53 mutant aggregation-associated cancer. This approach would open novel paths to treat cancer by treating it as a p53 protein aggregation-prone disease. 

## Figures and Tables

**Table 1:** Summary of recent studies on p53 Anti-Aggregate drugs.

p53 Anti-Aggregates	Findings
**a. Small stress molecules** (i) Arginine and analogues [28]	(i) Inhibit p53 mutant (R248Q) mimetic peptide aggregation in vitro, and (ii) inhibit p53 mutant lung cancer cells (H719, R248Q mutant), and breast cancer cells (SK-BR-3, R175H mutant) proliferation in vitro.
(ii) Acetylcholine chloride [27]	Inhibit p53 mutant (R248W) mimetic peptide aggregation in vitro.
**b. Designer peptide [22]**	Inhibit p53 mutant aggregation in vitro and in vivo in an ovarian cancer model
